# Mediator of DNA Damage Checkpoint 1 (MDC1) Contributes to High NaCl-Induced Activation of the Osmoprotective Transcription Factor TonEBP/OREBP

**DOI:** 10.1371/journal.pone.0012108

**Published:** 2010-08-11

**Authors:** Margarita Kunin, Natalia I. Dmitrieva, Morgan Gallazzini, Rong-Fong Shen, Guanghui Wang, Maurice B. Burg, Joan D. Ferraris

**Affiliations:** 1 Laboratory of Kidney and Electrolyte Metabolism, National Heart, Lung and Blood Institute, Bethesda, Maryland, United States of America; 2 Proteomics Core Facility, National Heart, Lung and Blood Institute, Bethesda, Maryland, United States of America; Virginia Commonwealth University, United States of America

## Abstract

**Background:**

Hypertonicity, such as induced by high NaCl, increases the activity of the transcription factor TonEBP/OREBP whose target genes increase osmoprotective organic osmolytes and heat shock proteins.

**Methodology:**

We used mass spectrometry to analyze proteins that coimmunoprecipitate with TonEBP/OREBP in order to identify ones that might contribute to its high NaCl-induced activation.

**Principal Findings:**

We identified 20 unique peptides from Mediator of DNA Damage Checkpoint 1 (MDC1) with high probability. The identification was confirmed by Western analysis. We used small interfering RNA knockdown of MDC1 to characterize its osmotic function. Knocking down MDC1 reduces high NaCl-induced increases in TonEBP/OREBP transcriptional and transactivating activity, but has no significant effect on its nuclear localization. We confirm six previously known phosphorylation sites in MDC1, but do not find evidence that high NaCl increases phosphorylation of MDC1. It is suggestive that MDC1 acts as a DNA damage response protein since hypertonicity reversibly increases DNA breaks, and other DNA damage response proteins, like ATM, also associate with TonEBP/OREBP and contribute to its activation by hypertonicity.

**Conclusions/Significance:**

MDC1 associates with TonEBP/OREBP and contributes to high NaCl-induced increase of that factor's transcriptional activity.

## Introduction

Although interstitial NaCl concentration normally is extremely high in the renal medulla, its cells are protected by accumulation of compatible organic osmolytes [1] and expression of heat shock proteins [2]. These protective responses are mediated by the transcription factor, Tonicity-responsive Enhancer/Osmotic Response Element-Binding Protein (TonEBP/OREBP, NFAT5) [3], [4]. High NaCl activates TonEBP/OREBP, which increases the transcription of genes whose protein products are involved in accumulation of organic osmolytes, including glycine betaine (BGT1, betaine/amino butyric acid transporter, SLC6A12), myo-inositol (SMIT, sodium-myo-inositol cotransporter, SLC5A3), glycerophosphocholine (Neuropathy Target Esterase, NTE, PNPLA6) and sorbitol (aldose reductase, AKR1B1) [5]. TonEBP/OREBP also increases transcription of Heat Shock Protein 70 (Hsp70-2, HSPA1B) [6]. High NaCl increases transcriptional activity of TonEBP/OREBP by several mechanisms. It causes TonEBP/OREBP to translocate to the nucleus [3], [4], increases the mRNA and protein abundance of TonEBP/OREBP [3], [4], increases activity of the TonEBP/OREBP transactivation domain (TAD) [7], and increases phosphorylation of TonEBP/OREBP [8]. Several different protein kinases are known to contribute to activation of TonEBP/OREBP, namely p38 MAP kinase (MAPK14) [9], tyrosine kinase Fyn (FYN) [9], protein kinase A (PKAcs, PRKACA) [10] and Ataxia Telangiectasia Mutated kinase (ATM) [11]. All contribute to high-NaCl-induced activation of TonEBP/OREBP, but no individual one is sufficient for full activation [5].

TonEBP/OREBP is part of a large protein complex [3]. Some of the other proteins in this complex are already known, based on coimmunoprecipitation with TonEBP/OREBP, including PKAcs [10], ATM [11], poly (ADP-ribose) polymerase 1 (PARP1) [12], heat shock protein 90 (HSP90, HSP90AA1) [12], activator protein 1 (AP-1, FOS/JUN) [13] and RNA Helicase A (RHA, DHX9)[12], [14], all of which have been shown to regulate activation of TonEBP/OREBP. Any additional proteins that physically associate with TonEBP/OREBP are candidates for participation in the transcriptional complex or signaling cascade. In the present study we used mass spectrometry to identify proteins that immunoprecipite in association with TonEBP/OREBP. We identify mediator of DNA damage checkpoint 1 (MDC1) as one of them, and find that it contributes to activation of TonEBP/OREBP. MDC1 is a DNA damage response protein, which is significant since hypertonicity reversibly increases DNA breaks and other DNA damage response proteins, like ATM [11], also associate with TonEBP/OREBP and contribute to its activation by hypertonicity.

## Results

### Identification by mass spectrometry of MDC1 as a TonEBP/OREBP-associated protein

**Figure 1:**
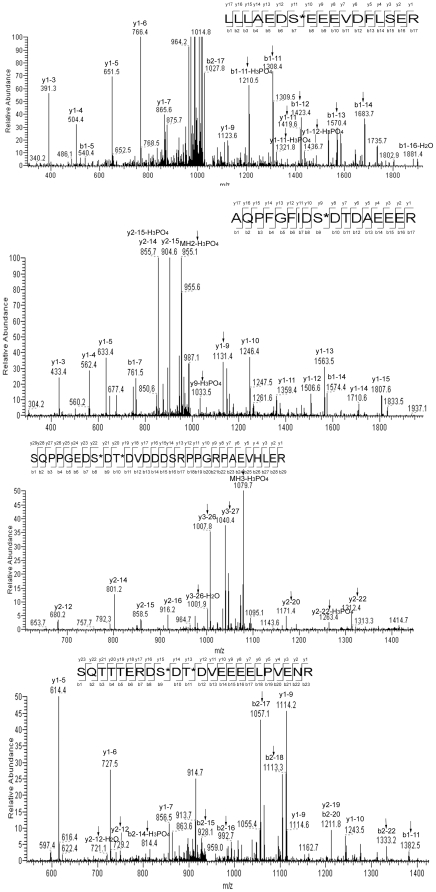
Identification of MDC1 in immunoprecipitates of TonEBP/OREBP-1-547-V5 by mass spectrometry. MS^2^ spectra of four MDC1 peptides. The arrows indicate ions that are site determining for phosphorylation.

To identify proteins that associate with and, thus, possibly regulate or support TonEBP/OREBP activity we immunoprecipitated stably transfected TonEBP/OREBP-1-547-V5 from nuclear and cytoplasmic extracts of HEK293 cells 2 hours after osmolality was changed from 300 to 200 or 500 mosmol/kg. We studied transfected TonEBP/OREBP because, like other transcription factors, the abundance of native TonEBP/OREBP protein is low. Also, the cells do not tolerate continuous over expression of the full length protein [12]. TonEBP/OREBP peptides were present in both nuclear and cytoplasmic fractions from cells at 300 mosmol/kg in 9 independent experiments, using either arginase or trypsin for proteolysis. There were up to 9 unique peptides in a single sample. MDC1 was also present in multiple independently prepared samples at both 200 and 500 mosmol/kg. Table 1 lists 20 different peptides from MDC1 that were identified with high probability. Representative spectra for four peptides are shown in Figure 1.

**Table 1:** MDC1 peptides identified by mass spectrometry.

Sequence	Charge	Xcorr
K.TPEPVVPTAPEPHPTTSTDQPVTP.K (aa 1607-1632)	3	4.3
R.AHEVGAQGGPPVAQVEQDLPISR.E (aa 619-643)	3	5.1
R.AMPVPTTPEFQSPVTTDQPISPEPITQPSCIKR.Q (aa 1690-1724)	3	5.3
R.ENLTDLVVDTDTLGESTQPQREGAQVPTGR.E (aa 642-673)	3	4.7
R.SSGKTPETLVPTAPKLEPSTSTDQPVTPEPTSQATR.G (aa 1398-1435)	3	5.8
R.SSVKTPEPVVPTAPELQPSTSTDQPVTSEPTSQVTR.G (aa 1152-1189)	3	7.4
R.SSVKTPEPVVPTAPELQPSTSTDQPVTSEPTYQATR.G (aa 1234-1271)	3	5.7
R.SSVKTPESIVPIAPELQPSTSR.N (aa 1562-1585)	3	4.1
R.SSVKTPETVVPTAPELQASASTDQPVTSEPTSR.T (aa 1480-1514)	3	6.1
K.KHQVSVEGTNQTDVK.A (aa 539-555)	3	3.9
K.TPETLVPTAPK.L (aa 1402-1414)	2	3.6
R.VGLPLLSPEFLLTGVLK.Q (aa 2054-2072)	2	4.2
R.IPATPVVIPMK.K (aa 337-349)	2	3.1
R.LLLAEDS*EEEVDFLSER.R (aa 161-179)	2	4.4
R.SQPPGEDS*DT*DVDDDSRPPGRPAEVHLER.A (aa 291-321)	3	3.3
R.SSVKT*PEPVVPTAPEPHPTTSTDQPVTPK.L (aa 1603-1633)	3	3.8
R.KSQLPAEGDAGAEWAAAVLKQER.A (aa 596-620)	3	6.1
R.TNMSSVKNPESTVPIAPELPPSTSTEQPVTPEPTSR.A (aa 1354-1391)	3	5.9
K.VLFTGVVDAR.G (aa 1894-1905)	2	2.7
R.DAEEDMPQR.V (aa 429-439)	2	3.2

Xcorr is the SEQUEST peptide cross-correlation score. XCorr values above 2.0 indicate a good identification. The charges on the ions are indicated.

*Identification by mass spectrometry of phosphorylation sites in MDC1.* We also identified phosphorylated amino acids in MDC1, namely S168, S299, T301, S329, S453, T455 (Table 2). High Ascores (27–153) confirm the identifications (Ascore >19 predicts >99% probability of correct identification). These phosphorylation sites were previously reported [15]–[18]. Also, S299, T301, and S453 were reported to be phosphorylated in vitro by recombinant CK2 [18].

**Table 2:** MDC1 phosphopeptides identified by mass spectrometry.

Sequence	Charge	Xcorr	Phosphosite	Ascore
R.LLLAEDS*EEEVDFLSER.R (aa 161-179)	2	4.37	S168	153.33
R.SQPPGEDS*DT*DVDDDSRPPGRPAEVHLER.A (aa 291-321)	3	3.33	S299	43.85
			T301	56.79
R.AQPFGFIDS*DTDAEEER.I (aa 320-338)	3	4.95	S329	38.66
R.SQTTTERDS*DT*DVEEEELPVENR.E (aa 444-468)	3	5.32	S453	26.79
			T455	42.62

Xcorr is peptide cross-correlation score. Ascore measures the probability of correct identity of the phosphorylation sites. Asterisks indicate phosphorylation of the preceding residues.

### Confirmation by immunoblot of identification of MDC1

**Figure 2:**
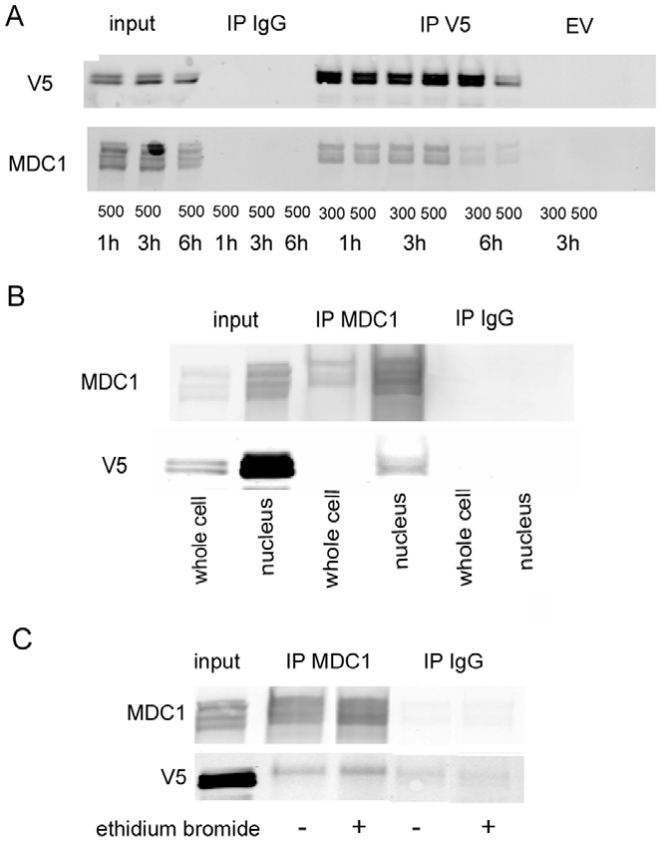
Confirmation by Western analysis of identification of MDC1 in immunoprecipitates of TonEBP/OREBP-1-547-V5. “Input” is the protein extract before immunoprecipitation. **A.** Osmolality bathing HEK 293 cells stably transfected with TonEBP/OREBP-1-547-V5 or empty vector-V5 (EV) was raised from 300 to 500 mosmol/kg by adding NaCl for 1, 3 and 6 h. Proteins were immunoprecipitated with rabbit IgG or anti-V5 antibodies and immunoblotted with anti-V5 or anti-MDC1 antibody. **B**. Osmolality bathing HEK 293 cells stably transfected with TonEBP/OREBP-1-547-V5 or empty vector-V5 was raised from 300 to 500 mosmol/kg by adding NaCl for 2 h. Proteins were immunoprecipitated from nuclear lysates with rabbit IgG or anti-MDC1 and immunoblotted with anti-MDC1 or anti-V5 antibody. **C**. 100 µg/ml ethidium bromide was added during immunoprecipitation with anti-MDC1 from nuclear extracts of the stably transfected HEK293 cells expressing TonEBP/OREBP-1-547-V5.

**Figure 3:**
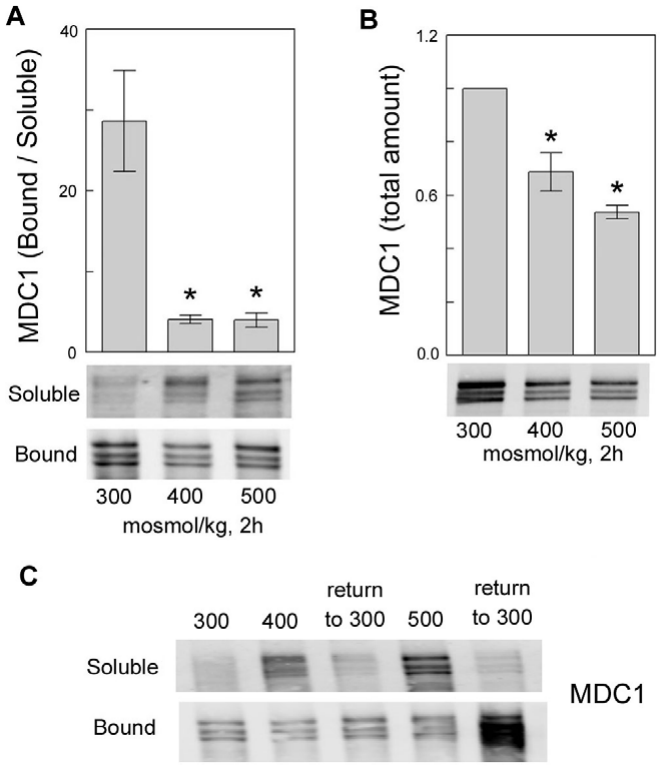
Effect of high NaCl on MDC1 abundance and distribution. HEK293 cells were exposed to media of the indicated total osmolalities (NaCl varied) for 2 h. **A.** High NaCl increases the abundance of MDC1 in the soluble fraction and decreases it in the chromatin-bound fraction. (n = 3, *P<0.05, t test). **B.** High NaCl decreases total MDC1 abundance (n = 3, *P<0.05, t test). **C.** After 2 h of exposure to high NaCl medium osmolality was decreased to 300 mosmol/kg for 30 min. MDC1 returns to the chromatin-bound fraction within that time (representative of 2 experiments).

**Figure 4:**
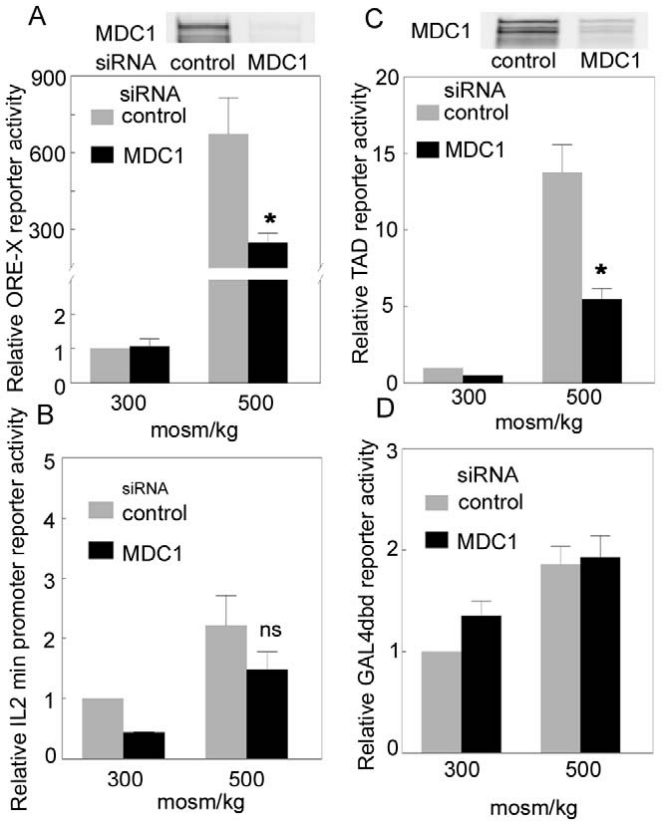
Effect on TonEBP/OREBP transcriptional and transactivating activities of knocking down MDC1 expression. **A.** HEK293 cells stably expressing an ORE-X reporter were transiently transfected with 25 nM of siRNA against MDC1 or control siRNA for 48 hours. Then, the osmolality of medium was changed to 500 mosm/kg by adding NaCl or kept at 300 mosmol/kg, and reporter activity was measured 24 h later. Knock down of MDC1 is shown in the upper panel. ORE-X reporter activity is relative to “control” at 300 mosmol/kg. **B.** Control for specificity for ORE. Same as (A) except using an IL2 min reporter (no ORE-X DNA element). **C.** Same as (A) except using HEK293 stably expressing the binary GAL4dbd TAD reporter and measuring luciferase activity 16 hours after adjusting osmolality. **D.** Control for specificity for TonEBP/OREBP TAD. Same as (C) except using HEK293 cells stably transfected with Gal4 DBD (no TAD). Mean ±SEM, *, P<0.01, n = 3.

Osmolality bathing HEK293 cells stably expressing TonEBP/OREBP-1-547-V5 or empty vector-V5 was increased to 500 mosmol/kg by adding NaCl for 1, 3 and 6 h. Anti-V5 immunoprecipitates from whole cell extracts were analyzed by Western blot. MDC1 is present in immunoprecipitates from cells transfected with TonEBP/OREBP-V5, but not from cells transfected with empty vector-V5 (Fig. 2A). MDC1 is a large protein; its bands run just above 250 kDa, which is the largest marker that we used. Further, immunoprecipitates with anti-MDC1 from nuclear extracts of the stably transfected HEK293 cells contain TonEBP/OREBP-V5 (Fig. 2B). The multiple bands in immunoblots of MDC1 (Figs. 2, 3 and 4) apparently represent alternatively spliced forms [19]. Interestingly, fewer bands of MDC1 are coimmunoprecititated with TonEBP/OREBP than are present in the input (Figure 2A). Perhaps, not all of the alternative splice variants associate with TonEBP/OREBP. In order to test whether the coimmunoprecipitation of MDC1 with TonEBP/OREBP reflects mutual binding to DNA rather than protein-protein interaction, we repeated anti-MDC1 immunoprecipitation in the presence of ethidium bromide (Fig. 2C), which intercalates DNA and disrupts protein-DNA interactions [20]. Since the presence of 100 µg/ml ethidium bromide does not interrupt the coimmunoprecipitation of MDC1 and TonEBP/OREBP-V5, we conclude that MDC1 and TonEBP/OREBP are physically associated by protein-protein interaction.

### High NaCl decreases total abundance of MDC1, but increases its soluble fraction

Increasing osmolality from 300 to 400 or 500 mosmol/kg by adding NaCl for 2 hours reduces chromatin-bound MDC1 and increases the soluble fraction (Fig. 3A), while decreasing the total abundance of MDC1 (Fig. 3B). However, when the osmolality is returned to 300 mosmol/kg, there is a large increase of chromatin-bound MDC1 (Fig. 3C). We also observed the same increase in HeLa cells (data not shown).

### Effect of MDC1 on TonEBP/OREBP transcriptional activity

We used an ORE-X luciferase reporter stably expressed in HEK293 cells to measure TonEBP/OREBP transcriptional activity. Raising osmolality from 300 to 500 mosmol/kg by adding NaCl increases the reporter activity more than 650-fold (Fig. 4A). We knocked MDC1 down with specific small interfering RNAs (siRNAs). 25 nM of MDC1 siRNA-1 decreases MDC1 abundance by approximately 80–90% (Fig. 4A). This specific siRNA reduces TonEBP/OREBP transcriptional activity by 69% compared to a non targeting control siRNA at 500 mosmol/kg, but has no significant effect at 300 mosmol/kg (Fig. 4A). Similarly, MDC1 siRNA-2 reduces TonEBP/OREBP transcriptional activity by 70% compared to the non targeting siRNA (mean of 2 independent experiments, data not shown). The fact that siRNAs targeting different parts of MDC1 have the same effect makes it unlikely that it is an off target effect. A stably transfected reporter that does not contain any ORE sequence served as control for specificity of the effect to TonEBP/OREBP. High NaCl does not increase activity of this control reporter and siRNA knockdown of MDC1 does not affect it significantly (Fig. 4B). We conclude that MDC1 contributes to high NaCl-induced increase of TonEBP/OREBP transcriptional activity.

### Effect of MDC1 on TonEBP/OREBP transactivating activity

We used a binary TonEBP/OREBP TAD luciferase reporter stably expressed in HEK293 cells to measure TonEBP/OREBP transactivating activity. Raising osmolality from 300 to 500 mosmol/kg by adding NaCl increases the reporter activity 13-fold (Fig. 4C). Knockdown of MDC1 by the specific siRNA-1 decreases TonEBP/OREBP transactivating activity by 60% at 500 mosmol/kg, but has no significant effect at 300 mosmol/kg (Fig. 4C). We also used a stable cell line with a reporter that does not contain the TonEBP/OREBP TAD as a control for specificity of the effect to TonEBP/OREBP. High NaCl does not increase activity of this control reporter and siRNA knockdown of MDC1does not affect it significantly (Fig. 4D). We conclude that MDC1 contributes to high NaCl-induced increase of TonEBP/OREBP transactivating activity.

### Lack of effect of knockdown of MDC1 on nuclear localization of TonEBP/OREBP

**Figure 5:**
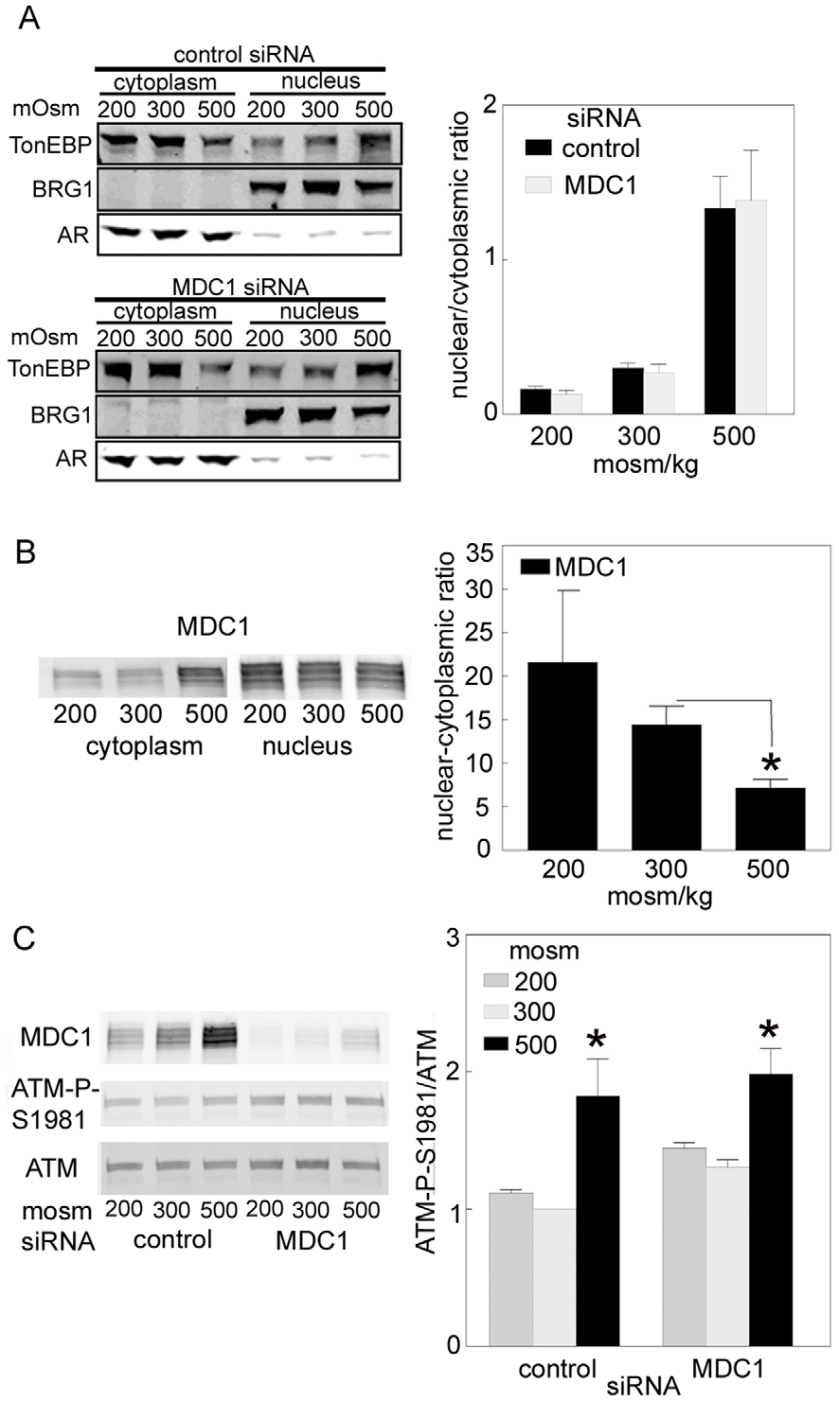
**A.** Lack of effect of siRNA-mediated knockdown of MDC1 on nuclear localization of TonEBP/OREBP. HEK 293 cells were transiently transfected with 25 nM MDC1 siRNA or control siRNA. 48 h later the osmolality of medium was changed to 200 or 500 mosmol/kg or kept at 300 mosmol/kg for 1 h. Nuclear and cytoplasmic extracts were prepared. TonEBP/OREBP nuclear/cytoplasmic ratio was calculated from its abundance in nuclear and cytoplasmic extracts. Antibodies to BRG1 and aldose reductase serve as controls for nuclear and cytoplasmic fractionation, respectively. **B**. High NaCl decreases the nuclear localization of MDC1. Osmolality was changed to 200 or 500 mosmol/kg or kept at 300 mosmol/kg for 1 h. Nuclear and cytoplasmic extracts were prepared. MDC1 nuclear/cytoplasmic ratio was calculated from its abundance in nuclear and cytoplasmic extracts. **C.** Lack of effect of siRNA-mediated knockdown of MDC1 on phosphorylation of ATM on Ser1981. As in (A), except that abundance of ATM was measured in whole cell extracts by Western analysis using a non-phosphospecific antibody and its phosphorylation on Ser1981 was measured using phosphospecific anti-ATM 1981S-P antibody. Results are presented as the ratio of phosphorylated to non phosphorylated ATM. (* P<.05, compared to 300 mosmol).

We measured TonEBP/OREBP protein by Western analysis of nuclear and cytoplasmic extracts in order to calculate its nuclear to cytoplasmic ratio Fig. 5A). The nuclear to cytoplasmic ratio of TonEBP/OREBP varies directly with NaCl concentration, and knocking down MDC1 with specific siRNA-1 has no significant effect on the ratio at 200, 300, or 500 mosmol/kg (Fig. 5A). Antibodies to BRG1 and aldose reductase serve as controls for nuclear and cytoplasmic fractionation, respectively (Fig. 5A).

### High NaCl decreases the nuclear to cytoplasmic ratio of soluble MDC1

We measured MDC1 protein by Western analysis of nuclear and cytoplasmic extracts in order to calculate its nuclear to cytoplasmic ratio (Fig. 5B). Note that the extraction method used does not extract chromatin-bound proteins.

### Lack of evidence for an effect of high NaCl on phosphorylation of MDC1

MDC1 becomes hyper phosphorylated in response to IR, as demonstrated by a retardation of its mobility in SDS-PAGE that is inhibited by phosphatase treatment of the protein extracts [19], [21]–[23]. The hyper phosphorylation is dependent on ATM and NBS1. We do not find gel retardation of MDC1 in response to high NaCl (Figs. 3 and 5B), but since the method is relatively insensitive, we cannot entirely exclude the possibility.

### Lack of effect of knockdown of MDC1 on ATM

ATM is a DNA damage response protein that is activated by phosphorylation on Ser1981 [24]. Suppression of MDC1 decreases ATM phosphorylation on Ser1981 in cells with DNA damaged by ionizing radiation (IR) [25]. Since high NaCl damages DNA and increases phosphorylation of ATM on Ser1981 [11], [26], we supposed that MDC1 might reduce the phosphorylation of ATM in response to high NaCl, like it does in response to IR. However, when we knock down MDC1 by 80–90% with the specific siRNA, there is no significant effect at any osmolality on phosphorylation of ATM on Ser1981 (Figure 5C).

## Discussion

TonEBP/OREBP is part of a large protein complex [3]. Many proteins in this complex are known contribute to regulation of TonEBP/OREBP, including PKAcs [10], PARP1 [12], Jun, Fos [13], RHA [14] and ATM [11]. In the present studies we find that MDC1 also is physically associated with TonEBP/OREBP (Figures 1, 2A, and 2B and Table 1) and contributes to its activation by high NaCl (Figures 4A and 4C). The complex containing TonEBP/OREBP and MDC1 apparently is preassembled since MDC1 coimmunoprecipitates with TonEBP/OREBP whether NaCl is elevated or not (Fig. 2A), as do other TonEBP/OREBP-associated proteins [12]. Evidently, high NaCl is not required for assembly of the large complex containing TonEBP/OREBP and the proteins that contribute to its osmotic regulation.

MDC1 works with H2AX to promote recruitment of repair proteins to the sites of DNA breaks [22]. High NaCl increases DNA breaks [27], [28] and alters chromosome structure [29]. Several other DNA damage response proteins also physically associate with TonEBP/OREBP, including DNA-PKcs (PRKDC) [12], Ku86 (XRCC5) [12], ATM [11], and PARP1 [12]. Further, ATM [11], PARP1[12], and MDC1 (present studies) all contribute to regulation of high NaCl-induced activation of TonEBP/OREBP. ATM is activated by high NaCl (determined from increased phosphorylation at S1981) and contributes to the high NaCl-induced increases of TonEBP/OREBP transcriptional activity [11], transactivating activity [11], and nuclear localization [30]. Although MDCI contributes to high NaCl-induced increase in TonEBP/OREBP transcriptional (Figure 4A) and transactivating activity (Figure 4C), it does not contribute to the nuclear localization (Figure 5A). ATM is a critical DNA repair protein, consistent with its activation in direct response to DNA damage. However, it may not be the DNA damage, itself, but the associated changes in chromatin that activate ATM in response to IR [31] and high NaCl.

High NaCl inhibits repair of DNA breaks caused by ultraviolet radiation (UV) [32], as well as the breaks caused by the high NaCl, itself [27]. Although those breaks are not repaired as long as NaCl remains high, they are rapidly repaired when the NaCl is lowered [27]. Further, the response of many damage response proteins to high NaCl-induced DNA breaks differs from their response to IR or UV. High NaCl, like IR or UV, activates ATM [11], but it does not induce γH2AX (phosphorylated histone H2AX) [32], [33] unless the level of salt is raised enough to kill the cells by apoptosis [33]. Also, high NaCl does not induce formation of MRN foci, composed of MRE11, RAD50 and NBS1 [33], and it reversibly inhibits induction of γH2AX by UV or IR [32].

MDC1 becomes partially immobilized to chromatin and recruits other DNA damage response proteins to the sites of DNA damage during repair of DNA breaks caused by IR [34]. In contrast, although high NaCl increases DNA breaks, it reduces the fraction of MDC1 bound to chromatin (Fig. 3A and C). A critical difference is that high NaCl inhibits DNA repair [27], inhibiting activation of DNA repair proteins, like γH2AX and reducing recruitment of the MRN complex to foci at the breaks [32]. We propose that exclusion of MDC1 from chromatin contributes to the inhibition by high NaCl of repair of DNA breaks. On the other hand, high NaCl-induced DNA breaks are rapidly repaired when NaCl is lowered [27]. The repair is accompanied by activation of DNA damage response proteins, like γH2AX, MRE11, and Chk1[27]. Accompanying that repair, the fraction of MDC1 bound to chromatin also increases dramatically (Figure 3C). We suggest that much of the increased binding of MDC1 occurs at the sites of DNA damage and contributes to their repair.

In so far as osmotic regulation of TonEBP/OREBP by MDC1 depends on their physical association and the transcriptional activity of TonEBP/OREBP depends on its binding to specific DNA elements [3], [4] the high NaCl-induced reduction of the fraction of MDC1 that is bound to chromatin (Figure 3) appears paradoxical. However, recall that MDC1 has at least two roles; it is both a DNA damage response protein and an osmotic regulator of TonEBP/OREBP. High NaCl inhibits DNA repair and reduces binding to DNA of repair proteins, including MDC1 (see above). Nevertheless, that leaves a substantial amount of MDC1 still bound to chromatin (Figure 3A), and the association of MDC1 with TonEBP/OREBP does remain intact (Figures 2A,B). We suggest that high NaCl-induced solubilization of chromatin-bound MDC1 could occur from pools not associated with TonEBP/OREBP, leaving MDC1 still associated with the TonEBP/OREBP bound to its specific DNA elements. On the other hand, regulation of TonEBP/OREBP by MDC1 does not necessarily require that they are bound together to DNA. TonEBP and MDC1 interact in the soluble fraction from the cells (Figure 2), and TonEBP/OREBP that is activated in the soluble fraction could subsequently bind to chromatin where it could activate transcription. An additional consideration is that release of MDC1 from chromatin near TonEBP/OREBP DNA elements could promote access of TonEBP/OREBP to those elements. The elements could be masked by proteins like MDC1, MRE11, Nbs1, and Rad51, that are released from chromatin when NaCl is elevated [35]. Also, release of MDC1 from chromatin could change chromatin conformation in a way specific for high NaCl.

In so far as DNA damage and/or changes in chromatin promote the role of ATM [5] and MDC1 in high NaCl-induced activation of TonEBP/OREBP, those changes might serve as sensors for hypertonicity. In this context it would be interesting to know whether DNA-PK and Ku86, which also physically associate with TonEBP/OREBP [12], have a similar role. Expression of Ku86, is already known to provide osmoprotection by an additional mechanism. It reduces high NaCl-induced chromosomal breakage, presumably by bridging broken DNA ends [35]. IR and UV do not directly activate TonEBP [11], so, any role of DNA damage in activating TonEBP/OREBP requires the context of hypertonicity.

In conclusion, we find the hypothesis attractive that high NaCl-induced increase in DNA breaks or alteration of chromatin structure provides a signal for the activation of TonEBP/OREBP through DNA damage response proteins, but we recognize that direct link between changes in DNA integrity and/or chromatin structure induced by high NaCl and activation of TonEBP/OREBP remains conjectural.

## Materials and Methods

### Plasmids and siRNA

Human TonEBP/OREBP cDNA clone KIAA0827 was a gift from Dr. Takahiro Nagase (Kazusa DNA Research Institute, Chiba, Japan). Sequence coding for amino acids 1–547 of KIAA0827 was cloned into expression vector pcDNA6V5-His (Invitrogen, Carlsbad, CA) to generate 1–547 V5-His as previously described [12]. The ORE-X luciferase reporter construct (used to measure TonEBP/OREBP transcriptional activity) contains two copies of human ORE-X [36] within a minimal IL-2 promoter [37] (hTonE-GL3, a gift from S. N. Ho, University of California at San Diego, La Jolla, CA). The control reporter (IL-2 min) does not contain any ORE sequence. The binary reporter system used to measure transactivating activity of TonEBP/OREBP consists of 1) a GAL4 reporter plasmid pFR-Luc (Stratagene, La Jolla, CA), containing five tandem repeats of the yeast GAL4 binding site (upstream activating sequence) upstream of a minimal promoter (TATATA) and the *P. pyralis* luciferase gene [7] and 2) GAL4dbd-TonEBP/OREBP, which contains the yeast GAL4 DNA binding domain (dbd) fused to sequence coding for amino acids 548–1531 of TonEBP/OREBP, which contain a NaCl-dependent TAD [7]. The control reporter, GAL4dbd, contains no TAD, but expresses the GAL4dbd (pFC2-dbd, Stratagene, La Jolla, CA).

We modified the siRNA previously used to knock down MDC1 [22], [38] to a synthetic dsRNA Dicer substrate to enhance the RNA interference potency and efficacy [39]. The control (nontargeting) siRNA (Integrated DNA Technologies, Coralville, IA) duplex sequences were: sense, 5′-Phos-UGAACCUGACCCAGGGGAGGGAGdTdT-3′ and antisense sequence 5′-AACUCCCUCCCCUGGGUCAGGUUCAUU-3′. The MDC1 siRNA (Integrated DNA Technologies, Coralville, IA) sequences were: sense for MDC1 siRNA1-5′- Phos-UCCAGUGAAUCCUUGAGGUGUAACGdTdT-3′, for MDC1 siRNA2 – 5′-Phos-GUCUCCCAGAAGACAGUGAUUAUCAdTdT-3′ and, antisense for siRNA1 – 5′-CGUUACACCUCAAGGAUUCACUGGAUU-3′, for siRNA2 – UGAUAAUCACUGUCUUCUGGGAGACUU-3′.

### Cell Culture and Treatment

HEK293 cells (American Type Culture Collection, ATTC, Manassas, VA) in passages 38–48 were cultured at 300 mosmol/kg in media recommended by ATCC. At experiment-specific time points, the medium was replaced with ones that were 300 mosmol/kg, 200 mosmol/kg (NaCl added to NaCl-free medium, Biofluids, Rockville, MD), or 500 mosmol/kg (NaCl added). HEK293 cells stably expressing TonEBP/OREBP 1-547 V5-His and stable HEK293 TonEBP/OREBP reporter cell lines were previously described [30], [40].

### Western Blot Analysis

Cells were lysed with a buffer containing 50 mM Tris-HCl, pH 8.0, 150 mM NaCl, 1% Triton X-100 for whole-cell extracts or with NE-PER (Nuclear and cytoplasmic Extraction Reagents; Pierce Biotechnology, Rockford, IL), according to supplier's instructions, for separate nuclear and cytoplasmic fractions. A protease inhibitor mixture (Roche Diagnostics, Indianapolis, IN) and phosphatase inhibitor cocktails I and II (Sigma-Aldrich, St. Louis, MO) were included in the lysis buffers. Proteins were separated on 4–12% Novex Tris- Glycine gels and transferred to nitrocellulose membranes (Invitrogen, Carlsbad, CA). Western blot analysis was performed according to instructions for the Odyssey Infrared Imaging System (Li-Cor, Lincoln, NE). In brief, nonspecific binding was blocked by incubating membranes for 1 h at 4°C with Odyssey Blocking Buffer diluted 1∶1 with PBS. Membranes then were incubated with rabbit anti-NFAT5 (TonEBP/OREBP) (Affinity BioReagents, Golden, CO), mouse anti-V5 (Invitrogen, Carlsbad, CA), rabbit anti-MDC1 (Bethyl Laboratories, Montgomery, TX), mouse anti-ATM (Santa Cruz Biotechnology, Santa Cruz, CA), goat anti-aldose reductase (Santa Cruz), mouse anti-BRG1 (Santa Cruz) or rabbit anti-P-ATM (Rockland Immunochemicals, Gilbertsville, PA) at 4°C overnight. After washing with 0.1% Tween-20 in PBS, blots were incubated with Alexa Fluor 680 goat anti-rabbit IgG or Alexa Fluor 780 goat anti-mouse IgG (Molecular Probes, Carlsbad, CA) for 1 h in the dark. Blots were visualized and quantitated by using a Li-Cor Odyssey Infrared Imager (Li-COR Biosciences, Lincoln, NE).

### Transfection and Luciferase Assays

25 nM of siRNA specific for MDC1 or a control siRNA was transfected with Lipofectamine 2000 (Invitrogen, Carlsbad, CA), according to supplier's instructions. 48 hours later the osmolality was either kept at 300 mosmol/kg or was increased to 500 mosmol/kg (NaCl added). Luciferase activity was measured with the Luciferase Assay System (Promega, Madison, WI) 24 h later for TonEBP/OREBP transcriptional activity (ORE-X reporter) or 16 h later for its transactivating activity (TAD reporter [7]). Total protein was measured with the BCA Protein Assay Kit (Pierce, Rockford, IL).

### Calculation of Nuclear/Cytoplasmic Ratios

The relative amounts of TonEBP/OREBP and MDC1 in the cytoplasmic and nuclear fractions and the nuclear/cytoplasmic ratio were calculated from their concentrations in cytoplasmic and nuclear extracts and the relative volumes of the extracts [41].

### Isolation of soluble and non-soluble proteins, Western blotting and Immunodetection

Cells were rinsed with phosphate-buffered saline (PBS), adjusted with NaCl to the same osmolality as the medium, then lysed with RIPA lysis buffer (50 mM Tris-HCl, 1% Igepal CA630, 150 mM NaCl, 1 mM EDTA, 1 mM NaF, 1 mM Na_3_VO_4_, and protease inhibitors (Roche Diagnostics)). The lysates were placed in ice for 10 min. Insoluble constituents were pelleted by centrifugation at 3,000 RCF. Supernatants containing proteins soluble in the RIPA buffer were transferred to separate tubes and protein concentration was measured using the BCA Protein Assay (Pierce, Rockford, IL). Insoluble pellets were boiled for 5 min in 40 µl of Laemmli Sample buffer to denature the proteins and release them from the pellet. After centrifugation at 15,000 RPM, the proteins in the supernatant were identified by Western analysis. Loading of the gels was normalized to equal masses of cells, calculated from the amounts of soluble proteins in the corresponding fractions. To calculate Bound to Soluble ratio for MDC1, the amount of MDC1 in the bound fraction (IF_bound_ (V_bound_ (total))/V_bound_(loaded)) was divided by amount of MDC1 in soluble fraction (IF_soluble_ (V_soluble_ (total))/V_soluble_ (loaded)), where V(loaded) is volume of the sample loaded on the gel, V(total) is volume of entire fraction and IF is Integral Fluorescence measured from corresponding band on immunoblot. To prepare whole cell extracts, cells were rinsed with phosphate-buffered saline (PBS), adjusted with NaCl to the same osmolality as the medium, then lysed with RIPA buffer. 3X Laemmli Sample buffer was added to the lysates and samples were boiled for 5 minutes. Samples were loaded according to protein concentration measured before addition of Laemmli buffer and equal loading was verified by Coomassie Blue staining of the gels.

### Immunoprecipitation and sample preparation for mass spectrometry

HEK 293 cells stably transfected with TonEBP/OREBP 1-547 V5-His were grown in 15-cm dishes. Osmolality was increased to 500 mosmol/kg or decreased to 200 mosmol/kg by adjusting NaCl for 2 h, then nuclear and cytoplasmic extracts were prepared with Ne-PER (Pierce, Rockford, IL) according to the supplier's instructions, with added protease inhibitor cocktail (Roche Applied Science, Germany) and phosphatase inhibitor cocktail I and II (Sigma, St. Louis, MO). Extracts were pre-cleared for 1 hour with rabbit IgG biotin-conjugated antibodies (Santa Cruz Biotechnology, Carlsbad, CA) on Dynabeads (Invitrogen. Pre-cleared supernatants were incubated overnight with rabbit anti-V5 biotin-conjugated antibodies (ICL, Inc, Newberg, OR) on Dynabeads. Beads were washed 3 times with buffer containing 50 mM Tris-HCl, pH 8.0, 150 mM NaCl, 1% Triton X-100, then 3 times with phosphate buffered saline containing 1% Triton X-100. Both buffers included phosphatase and protease inhibitor cocktails. The beads were resuspended in 6 M guanidine-HCl/50 mM NH_4_HCO_3_, to denature the proteins and elute them from the beads. Proteins were reduced with 100 mM DTT for 1 h at 56°C, then alkylated using 100 mM iodoacetamide for 1 h at room temperature in the dark. The sample buffer was exchanged to 50 mM NH_4_HCO_3_ for trypsin digestion and to 100 mM Tris-HCl, 10 mM CaCl_2_, pH 7.6 for endoproteinase Arg-C digestion, using Amicon Ultra Centrifugal Filter Devices (Millipore Corporation Billerica, MA). Samples were digested with 1∶50 wt/wt trypsin (Promega, Madison, WI) or endoproteinase Arg-C (Roche Applied Science, Germany) overnight at 37°C. The resulting peptides were desalted using a 1-ml hydrophilic-lipophilic-balanced (HLB) cartridge (Oasis, Milford, MA), followed by volume reduction *in vacuo*. Each sample was subsequently resuspended in 50 µl of 5% acetic acid, pH 2.5–3.0, then loaded onto an IMAC column (Pierce, Rockford, IL) for phosphopeptide enrichment. Peptides were incubated with the Ga 3+ resin in the column for 20 min with gentle agitation every 5 min, then washed and eluted as recommended by the supplier. Both flow through and eluate were analyzed. Samples were dried *in vacuo*, resuspended in 1% formic acid, and desalted with C18 Ziptips (Millipore Corporation, Billerica, MA) before analysis by MS. Immunoprecipitation of MDC1 utilized anti-MDC1 (Bethyl Laboratories, Montgomery, TX) on A/G PLUS-agarose beads.

### Mass spectrometry

Peptides in the flow-through fractions from IMAC columns and eluates from the columns were analyzed on an Agilent 1100 nanoflow system (Agilent Technologies, Palo Alto, CA) LC connected to a Finnigan LTQ mass spectrometer (Thermo Electron, San Jose, CA) equipped with a nanoelectrospray ion source. MS spectra were analyzed with the SEQUEST search algorithm in BIOWORKS software (Thermo Electron), to identify peptides. Peak masses were searched against the most current version of the Human Refseq Database (National Center for Biotechnology Information) and its reversed complement with the following parameters: fixed carbamidomethylation of Cys; variable phosphorylation of Ser, Thr, and Tyr. We used the target-decoy approach for matches to the MS^2^ spectra with a concatenated database that includes both forward and reversed sequences. The target-decoy approach estimates false discovery rates based on the principle that incorrect spectral matches have an equal probability of occurring in either the forward (“target”) or reversed (“decoy”) database. We used PhosphoPIC [42] to select for minimum cross-correlation (Xcorr) settings high enough to reduce the target false positive rate to less than 5%. All *.dta and *.out files from the SEQUEST search were included in the filtered dataset. The (XCorr) filter was automatically adjusted for each individual charge state (+1, +2, +3) to meet predetermined target false discovery rate, based on the number of allowable random matches from the reversed “decoy” database. We used Ascore (http://Ascore.med.harvard.edu) [43] to estimate the probability that the phosphorylation sites are correctly identified, based on the presence and intensity of site-determining ions in MS/MS spectra. Ascore >19 predicts >99% probability of correct identification.

### Statistical Analysis

Data were compared by t test or for multiple comparisons by repeated measures ANOVA, followed by Bonferroni multiple comparisons test. Normalized data were transformed prior to statistical analysis. Differences were considered significant for p≤0.05.

## References

[1] Bagnasco S, Balaban R, Fales HM, Yang YM, Burg M (1986). Predominant osmotically active organic solutes in rat and rabbit renal medullas.. J Biol Chem.

[2] Rauchman MI, Pullman J, Gullans SR (1997). Induction of molecular chaperones by hyperosmotic stress in mouse inner medullary collecting duct cells.. Am J Physiol.

[3] Miyakawa H, Woo SK, Dahl SC, Handler JS, Kwon HM (1999). Tonicity-responsive enhancer binding protein, a Rel-like protein that stimulates transcription in response to hypertonicity.. Proc Natl Acad Sci U S A.

[4] Ko BC, Turck CW, Lee KW, Yang Y, Chung SS (2000). Purification, identification, and characterization of an osmotic response element binding protein.. Biochem Biophys Res Commun.

[5] Burg MB, Ferraris JD, Dmitrieva NI (2007). Cellular response to hyperosmotic stresses.. Physiol Rev.

[6] Woo SK, Lee SD, Na KY, Park WK, Kwon HM (2002). TonEBP/NFAT5 stimulates transcription of HSP70 in response to hypertonicity.. Mol Cell Biol.

[7] Ferraris JD, Williams CK, Persaud P, Zhang Z, Chen Y, Burg MB (2002). Activity of the TonEBP/OREBP transactivation domain varies directly with extracellular NaCl concentration.. Proc Natl Acad Sci U S A.

[8] Dahl SC, Handler JS, Kwon HM (2001). Hypertonicity-induced phosphorylation and nuclear localization of the transcription factor TonEBP.. Am J Physiol Cell Physiol.

[9] Ko BC, Lam AK, Kapus A, Fan L, Chung SK, Chung SS (2002). Fyn and p38 signaling are both required for maximal hypertonic activation of the OREBP/TonEBP.. J Biol Chem.

[10] Ferraris JD, Persaud P, Williams CK, Chen Y, Burg MB (2002). cAMP-independent role of PKA in tonicity-induced transactivation of tonicity-responsive enhancer/osmotic response element-binding protein.. Proc Natl Acad Sci U S A.

[11] Irarrazabal CE, Liu JC, Burg MB, Ferraris JD (2004). ATM, a DNA damage-inducible kinase, contributes to activation by high NaCl of the transcription factor TonEBP/OREBP.. Proc Natl Acad Sci U S A.

[12] Chen Y, Schnetz MP, Irarrazabal CE, Shen RF, Williams CK, Burg MB, Ferraris JD (2007). Proteomic identification of proteins associated with the osmoregulatory transcription factor TonEBP/OREBP: functional effects of Hsp90 and PARP-1.. Am J Physiol Renal Physiol.

[13] Irarrazabal CE, Williams CK, Ely MA, Birrer MJ, Garcia-Perez A (2008). Activator protein-1 contributes to high NaCl-induced increase in tonicity-responsive enhancer/osmotic response element-binding protein transactivating activity.. J Biol Chem.

[14] Colla E, Lee SD, Sheen MR, Woo SK, Kwon HM (2005). TonEBP is inhibited by RNA helicase A via interaction involving the E'F loop.. Biochem J.

[15] Beausoleil SA, Jedrychowski M, Schwartz D, Elias JE, Villen J, Li J (2004). Large-scale characterization of HeLa cell nuclear phosphoproteins.. Proc Natl Acad Sci U S A.

[16] Olsen JV, Blagoev B, Gnad F, Macek B, Kumar C (2006). Global, in vivo, and site-specific phosphorylation dynamics in signaling networks.. Cell.

[17] Melander F, Bekker-Jensen S, Falck J, Bartek J, Mailand N (2008). Phosphorylation of SDT repeats in the MDC1 N terminus triggers retention of NBS1 at the DNA damage-modified chromatin.. J Cell Biol.

[18] Spycher C, Miller ES, Townsend K, Pavic L, Morrice NA (2008). Constitutive phosphorylation of MDC1 physically links the MRE11-RAD50-NBS1 complex to damaged chromatin.. J Cell Biol.

[19] Goldberg M, Stucki M, Falck J, D'Amours D, Rahman D (2003). MDC1 is required for the intra-S-phase DNA damage checkpoint.. Nature.

[20] Lai JS, Herr W (1992). Ethidium bromide provides a simple tool for identifying genuine DNA-independent protein associations.. Proc Natl Acad Sci U S A.

[21] Lou Z, Minter-Dykhouse K, Wu X, Chen J (2003). MDC1 is coupled to activated CHK2 in mammalian DNA damage response pathways.. Nature.

[22] Stewart GS, Wang B, Bignell CR, Taylor AM, Elledge SJ (2003). MDC1 is a mediator of the mammalian DNA damage checkpoint.. Nature.

[23] Xu X, Stern DF (2003). NFBD1/KIAA0170 is a chromatin-associated protein involved in DNA damage signaling pathways.. J Biol Chem.

[24] Bakkenist CJ, Kastan MB (2003). DNA damage activates ATM through intermolecular autophosphorylation and dimer dissociation.. Nature.

[25] Mochan TA, Venere M, DiTullio RA, Halazonetis TD (2003). 53BP1 and NFBD1/MDC1-Nbs1 function in parallel interacting pathways activating ataxia-telangiectasia mutated (ATM) in response to DNA damage.. Cancer Res.

[26] Dmitrieva NI, Burg MB, Ferraris JD (2005). DNA damage and osmotic regulation in the kidney.. Am J Physiol Renal Physiol.

[27] Dmitrieva NI, Cai Q, Burg MB (2004). Cells adapted to high NaCl have many DNA breaks and impaired DNA repair both in cell culture and in vivo.. Proc Natl Acad Sci U S A.

[28] Kultz D, Chakravarty D (2001). Hyperosmolality in the form of elevated NaCl but not urea causes DNA damage in murine kidney cells.. Proc Natl Acad Sci U S A.

[29] Tong EH, Guo JJ, Xu SX, Mak K, Chung SK (2009). Inducible nucleosome depletion at OREBP-binding-sites by hypertonic stress.. PLoS One.

[30] Zhang Z, Ferraris J, Irarrazabal CE, Dmitireva NI, Park JH (2005). Ataxia-telangiectasia mutated (ATM), a DNA damage-inducible kinase, contributes to high NaCl-induced nuclear localization of the transcription factor TonEBP/OREBP.. Am J Physiol Renal Physiol.

[31] Kim YC, Gerlitz G, Furusawa T, Catez F, Nussenzweig A (2009). Activation of ATM depends on chromatin interactions occurring before induction of DNA damage.. Nat Cell Biol.

[32] Dmitrieva NI, Bulavin DV, Burg MB (2003). High NaCl causes Mre11 to leave the nucleus, disrupting DNA damage signaling and repair.. Am J Physiol Renal Physiol.

[33] Dmitrieva NI, Burg MB (2008). Analysis of DNA breaks, DNA damage response, and apoptosis produced by high NaCl.. Am J Physiol Renal Physiol.

[34] Lukas C, Melander F, Stucki M, Falck J, Bekker-Jensen S (2004). Mdc1 couples DNA double-strand break recognition by Nbs1 with its H2AX-dependent chromatin retention.. EMBO J.

[35] Dmitrieva NI, Celeste A, Nussenzweig A, Burg MB (2005). Ku86 preserves chromatin integrity in cells adapted to high NaCl.. Proc Natl Acad Sci U S A.

[36] Ferraris JD, Williams CK, Ohtaka A, Garcia-Perez A (1999). Functional consensus for mammalian osmotic response elements.. Am J Physiol.

[37] Trama J, Lu Q, Hawley RG, Ho SN (2000). The NFAT-related protein NFATL1 (TonEBP/NFAT5) is induced upon T cell activation in a calcineurin-dependent manner.. J Immunol.

[38] Lou Z, Chen J (2004). Use of siRNA to study the function of MDC1 in DNA damage responses.. Methods Mol Biol.

[39] Kim DH, Behlke MA, Rose SD, Chang MS, Choi S (2005). Synthetic dsRNA Dicer substrates enhance RNAi potency and efficacy.. Nat Biotechnol.

[40] Zhou X, Ferraris JD, Burg MB (2006). Mitochondrial reactive oxygen species contribute to high NaCl-induced activation of the transcription factor TonEBP/OREBP.. Am J Physiol Renal Physiol.

[41] Ferraris JD, Burg MB (2007). Tonicity-regulated gene expression.. Methods Enzymol.

[42] Hoffert JD, Wang G, Pisitkun T, Shen RF, Knepper MA (2007). An automated platform for analysis of phosphoproteomic datasets: application to kidney collecting duct phosphoproteins.. J Proteome Res.

[43] Beausoleil SA, Villen J, Gerber SA, Rush J, Gygi SP (2006). A probability-based approach for high-throughput protein phosphorylation analysis and site localization.. Nat Biotechnol.

